# *N*-(3,5-Di­chloro-4-meth­oxy­phen­yl)acetamide

**DOI:** 10.1107/S241431462600502X

**Published:** 2026-05-22

**Authors:** Rao M. Uppu, Sindhu V. Bai, Patrick F. Mensah, Guoqiang Li, Frank R. Fronczek

**Affiliations:** a Department of Environmental Toxicology Southern University and A&M College,Baton Rouge Louisiana 70813 USA; b Department of Mechanical Engineering Southern University and A&M College,Baton Rouge Louisiana 70813 USA; c Department of Mechanical and Industrial Engineering Louisiana State University,Baton Rouge Louisiana 70803 USA; d Department of Chemistry Louisiana State University,Baton Rouge Louisiana 70803 USA; University of Aberdeen, United Kingdom

**Keywords:** crystal structure, cellular oxidants, chlorinated methacetin, hypo­chlorous acid-hypochlorite, mitotoxicity, protonophores

## Abstract

The title compound, C_9_H_9_Cl_2_NO_2_, crystallizes in the triclinic space group *P*1 with six mol­ecules in the asymmetric unit. Structure determination at 100 K from a two-component twin crystal shows that the six crystallographically independent mol­ecules adopt closely similar conformations. The di­chloro­phenyl and acetamide planes form a dihedral angle of *ca*. 10.0° (mean of six mol­ecules), while the meth­oxy substituent is oriented nearly orthogonal to the aromatic ring (mean dihedral angle = 88.5°). In the extended structure, the mol­ecules are linked by N—H⋯O hydrogen bonds into infinite chains extending along [110], giving rise to three independent motifs of alternating mol­ecules (*ABABAB*⋯, *CDCDCD*⋯, and *EFEFEF*⋯). Numerous weak C—H⋯O and C—H⋯Cl hydrogen bonds consolidate the packing.

## Abstract 2

The title compound crystallizes as a two-component twin in space group *P*1 with *Z*′ = 6, exhibiting packing-driven conformational multiplicity and N—H⋯O hydrogen-bonded chains propagating along [110].

## Graphical abstract

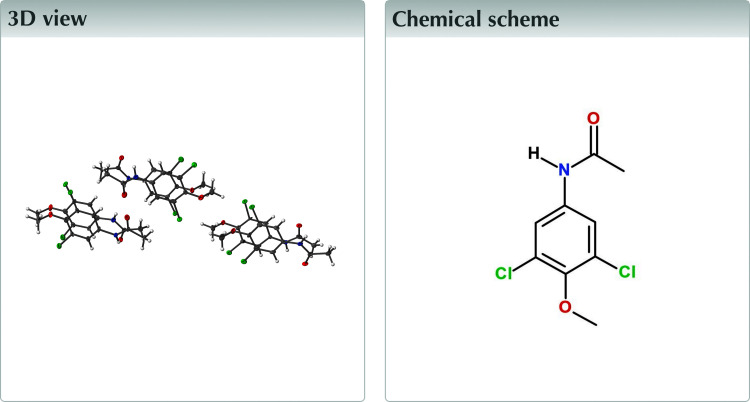

## Structure description

A chlorinated derivative of methacetin, *N*-(3,5-di­chloro-4-meth­oxyphen­yl)acetamide, can be formed during hypo­chlorous acid (HOCl)/hypochlorite (ClO^−^)-mediated oxid­ation; such electrophilic chlorination reactions are of potential relevance to xenobiotic biotransformation and mineralization (Acero *et al.*, 2010 ▸; Hines *et al.*, 2026 ▸). Methacetin belongs to the class of alk­oxy­acetanilides that includes phenacetin (C_10_H_13_NO_2_), an early synthetic analgesic and anti­pyretic introduced in the late 19th Century following the clinical use of acetanilide (Anti­febrin; C_8_H_9_NO), whose use declined due to toxicity concerns (Merck, 1899 ▸). Methacetin itself was not used as an analgesic–anti­pyretic but has been employed since the 1990s in liver function testing *via* the [^13^C-meth­yl]-methacetin breath test, which monitors CYP-mediated oxidative O-de­alkyl­ation (primarily by CYP1A2) through measurement of ^13^CO_2_ in exhaled breath (Buechter & Gerken, 2022 ▸; Gairing *et al.*, 2022 ▸; Santol *et al.*, 2024 ▸).

**Figure 1:**
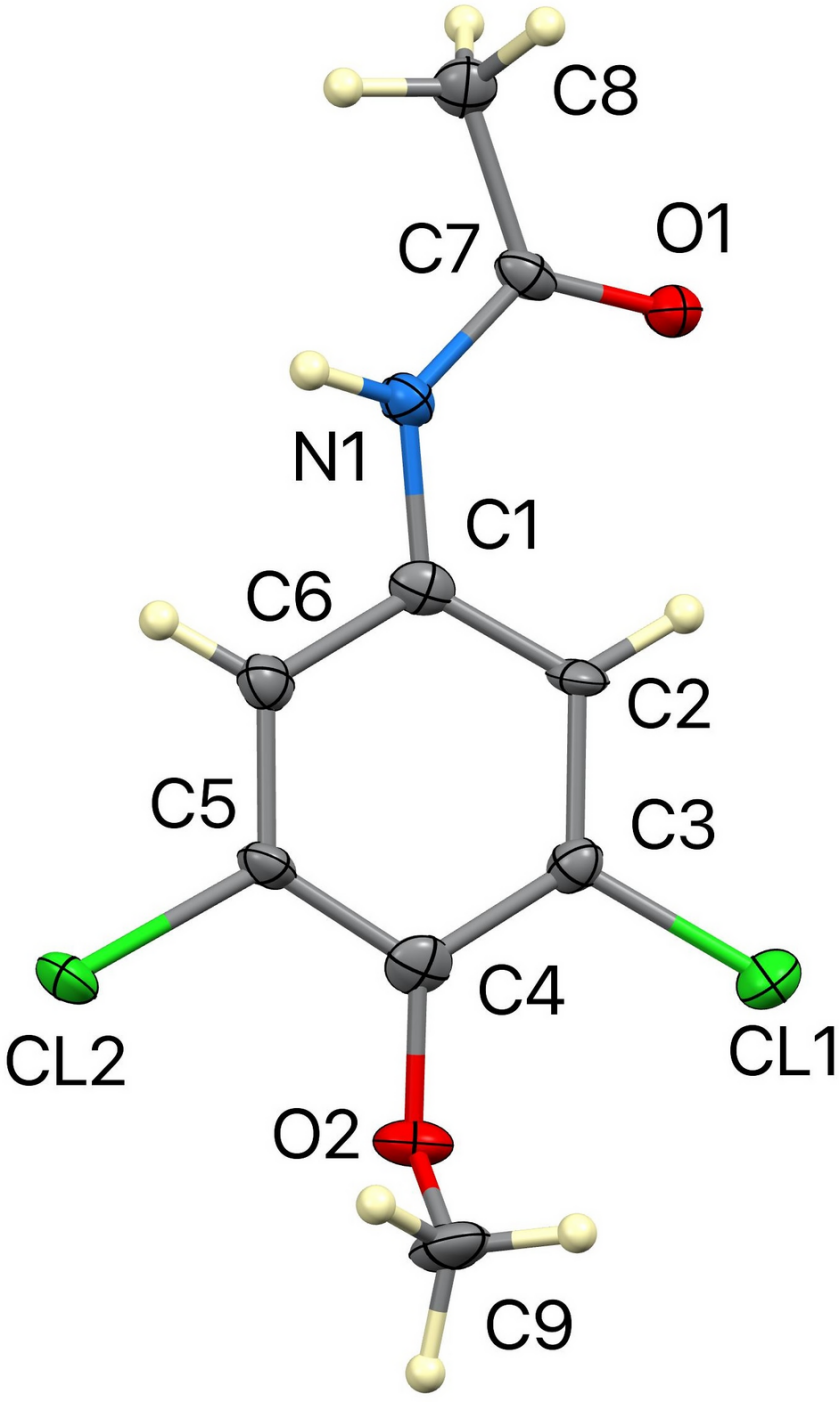
The mol­ecular structure of one of the six mol­ecules of (**I**), with atom labeling and displacement ellipsoids drawn at the 50% probability level.

In this context, and in view of the susceptibility of methacetin and related compounds to biotransformation by cellular oxidants such as HOCl/ClO^−^ (Hines *et al.*, 2026 ▸) and per­oxy­nitrite (ONOOH)/per­oxy­nitrite (ONOO^−^) (Hines *et al.*, 2025 ▸), as well as the potential formation of protonophoric metabolites, including *N*-(3,5-di­chloro-4-meth­oxyphen­yl)acetamide *via* oxidative O-de­alkyl­ation (Uppu & Fronczek, 2026*a* ▸,*b* ▸), we have synthesized and structurally characterized the title compound, C_9_H_9_Cl_2_NO_2_, (**I**) (Fig. 1 ▸) (Bai *et al.*, 2026 ▸).

The asymmetric unit of (**I**) contains six independent mol­ecules (*Z*′ = 6), an unusually high value for a compound of this size, indicative of packing-stabilized conformational multiplicity. Only about 0.057% of structures in the Cambridge Structural Database (Groom *et al.*, 2016 ▸) have *Z*′ = 6. Each mol­ecule comprises a dichlorinated aromatic ring bearing meth­oxy and acetamide substituents. The mean dihedral angle between the aromatic ring and the acetamide plane is approximately 10°, indicating near coplanarity, whereas the meth­oxy group is oriented nearly orthogonal to the ring (*ca*. 88°). Bond distances and angles are within expected ranges, with C—Cl distances averaging *ca*. 1.73 Å.

**Figure 2:**
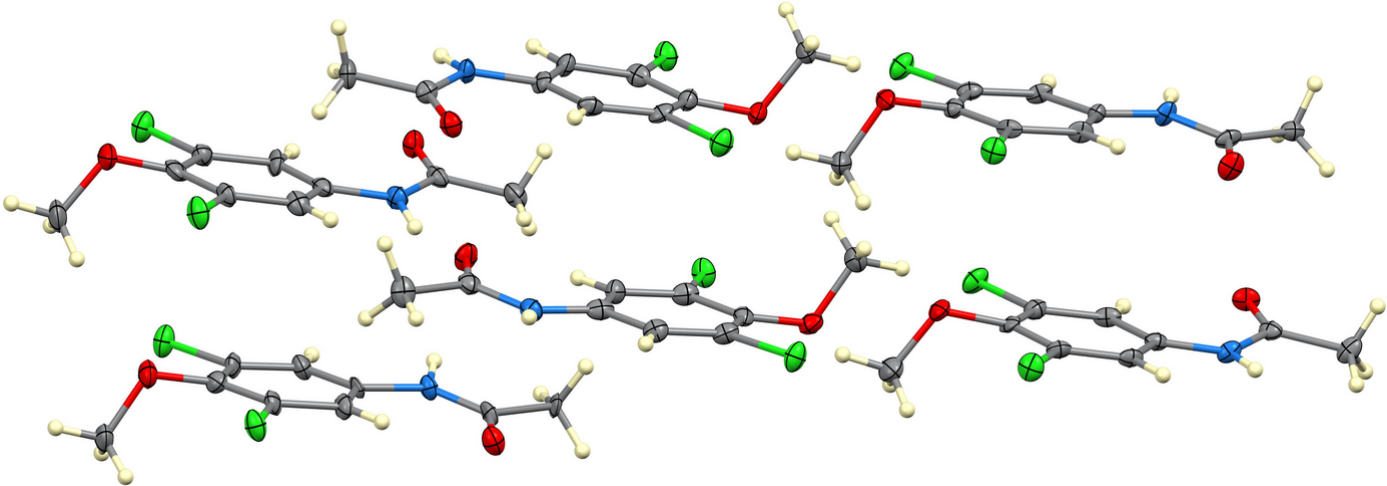
View of the six crystallographically independent mol­ecules in the asymmetric unit of (**I**), illustrating the conformational similarity among the independent mol­ecules.

**Figure 3:**
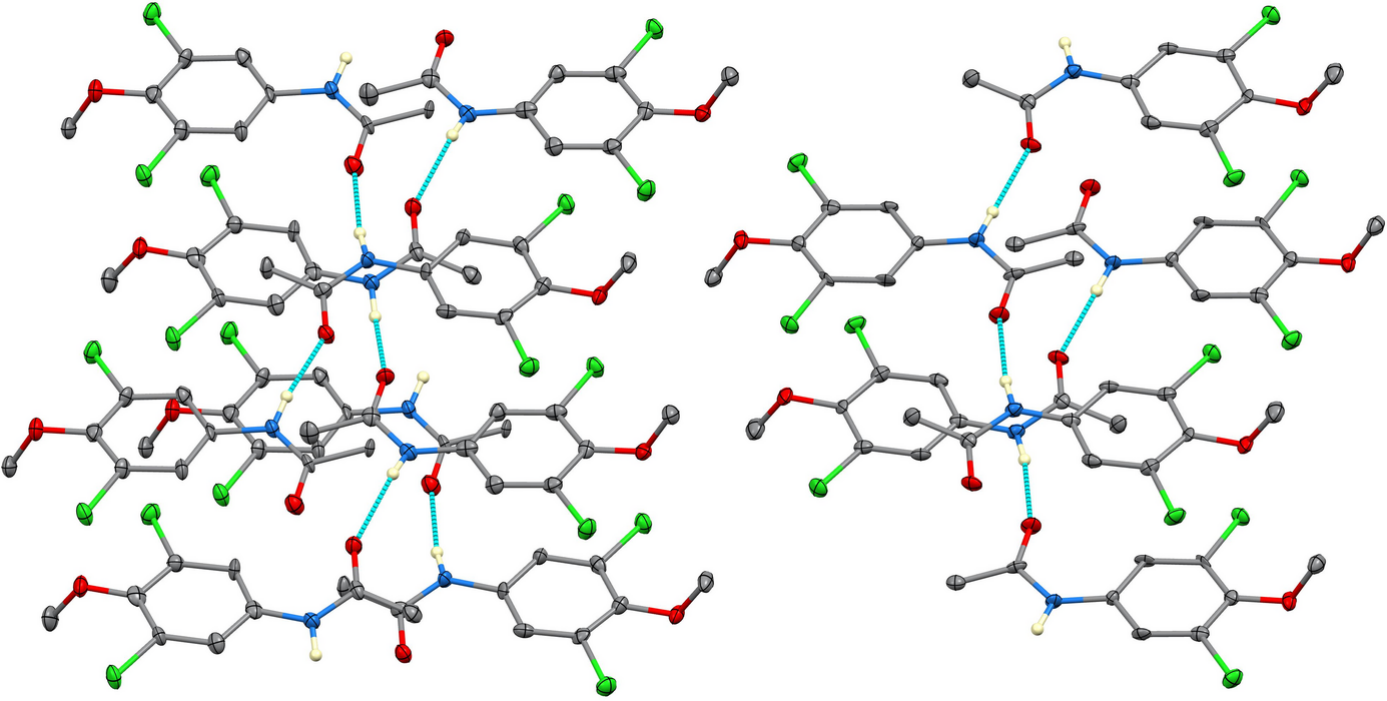
Partial packing diagram of (**I**) showing the N—H⋯O hydrogen-bonded chains propagating along the [1
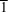
0] direction.

**Figure 4:**
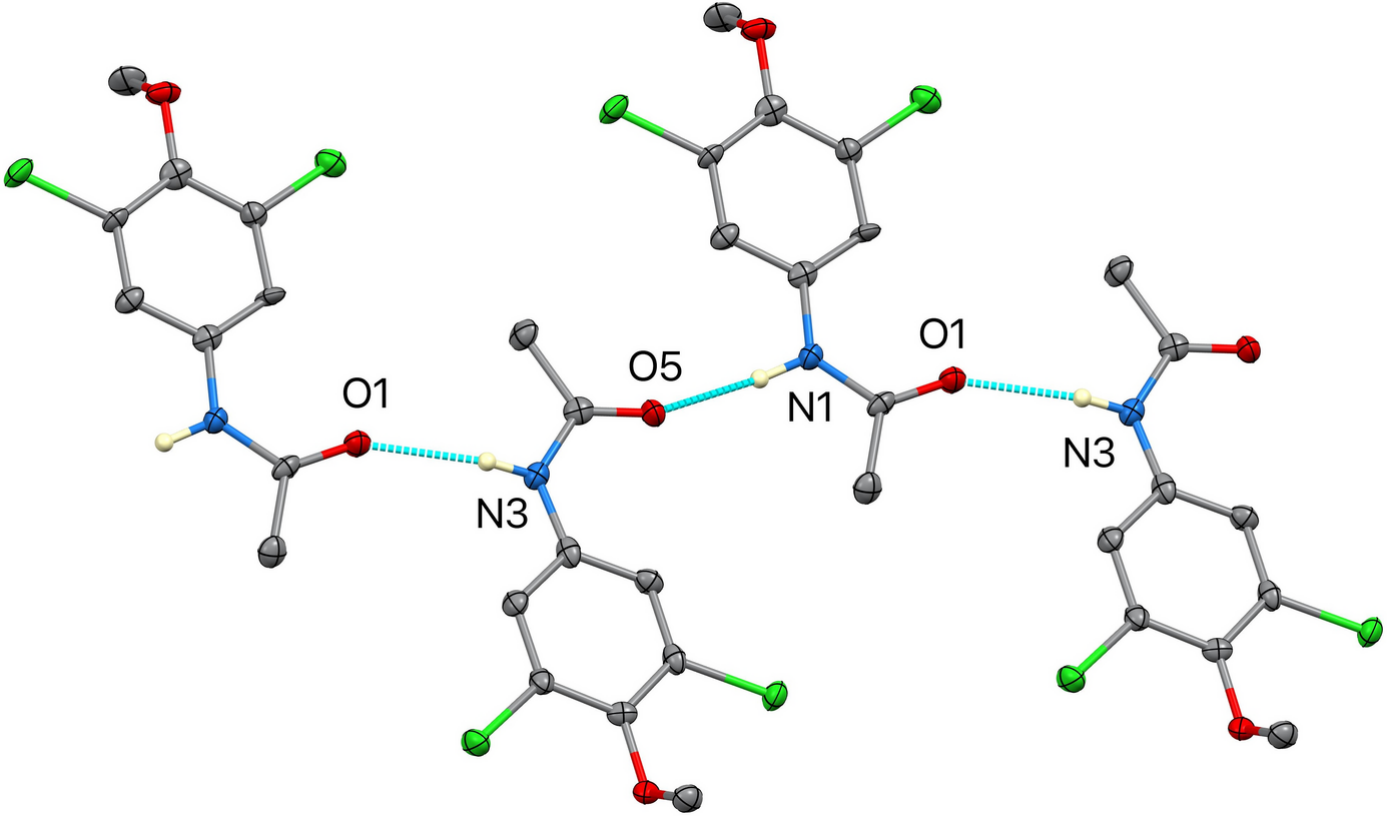
Detail of one hydrogen-bonded chain in (**I**), highlighting the alternating mol­ecular arrangement within the chain motif.

**Figure 5:**
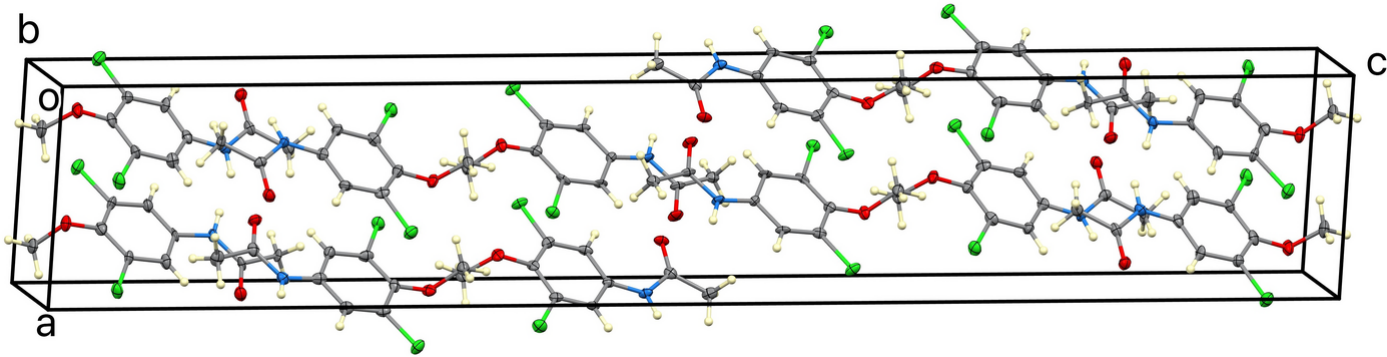
View of the crystal packing of (**I**) approximately along the *b* axis, showing the arrangement of mol­ecules within the unit cell.

In the extended structure (Figs. 2–5 ▸ ▸ ▸ ▸), N—H⋯O hydrogen bonds link the mol­ecules into chains extending along the [1
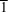
0] direction. Three distinct but structurally analogous chain motifs (*ABABAB*⋯, *CDCDCD*⋯ and *EFEFEF*⋯) arise from the six independent mol­ecules. These inter­actions, together with longer C—H⋯O and C—H⋯Cl contacts (Table 1 ▸) further consolidate the crystal packing. The presence of multiple independent mol­ecules and twinning reflects subtle packing-driven stabilization of quasi-equivalent conformers rather than large intrinsic conformational differences.

## Synthesis and crystallization

The title compound was synthesized by acetyl­ation of 3,5-di­chloro-4-meth­oxy­aniline with acetic anhydride in acetic acid as solvent. The anhydride, typically taken in 20% molar excess (6.4 mmol), was reacted with 5.3 mmol of the aniline derivative in 10 ml of acetic acid, and the mixture was stirred using a magnetic stir bar for 1 h at room temperature. The solvent was then evaporated, and the product was purified by recrystallization from ethanol solution. Crystals of (**I**) suitable for X-ray diffraction were obtained by slow cooling and evaporation of a hot ethano­lic solution under ambient conditions.

## Refinement

Crystallographic and refinement data are summarized in Table 2 ▸. All H atoms were located in difference maps and those on C were thereafter treated as riding in geometrically idealized positions with C—H distances 0.95 Å for phenyl and 0.98 Å for methyl. The coordinates of the N-bound hydrogen atoms were refined with N—H distances restrained to be equal. *U*_iso_(H) values were assigned as 1.2*U*_eq_ for the attached atom (1.5 for meth­yl). The crystal chosen for data collection was found to be a two-component pseudomerohedral twin by twofold rotation around (001) (real space) or [0
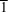
2] (reciprocal space). The twin law is (–1 0 0 / 0 −1 0 / 0 −1 1), and the twin domains refined to 0.7468 (4)/0.2532 (4).

## Supplementary Material

Crystal structure: contains datablock(s) I. DOI: 10.1107/S241431462600502X/hb4561sup1.cif

Structure factors: contains datablock(s) I. DOI: 10.1107/S241431462600502X/hb4561Isup2.hkl

Supporting information file. DOI: 10.1107/S241431462600502X/hb4561Isup3.cml

CCDC reference: 2536293

Additional supporting information:  crystallographic information; 3D view; checkCIF report

## Figures and Tables

**Table 1:** Hydrogen-bond geometry (Å, °)

*D*—H⋯*A*	*D*—H	H⋯*A*	*D*⋯*A*	*D*—H⋯*A*
N1—H1*N*⋯O5	0.90 (2)	2.08 (2)	2.932 (4)	158 (4)
C2—H2⋯O1	0.95	2.29	2.885 (5)	120
C6—H6⋯O5	0.95	2.63	3.406 (6)	140
C8—H8*B*⋯Cl8	0.98	2.92	3.842 (5)	157
C9—H9*B*⋯O4	0.98	2.52	3.437 (6)	157
N5—H5*N*⋯O11^i^	0.90 (2)	2.03 (2)	2.922 (4)	174 (5)
C38—H38⋯O9	0.95	2.29	2.878 (6)	120
C44—H44*C*⋯Cl10^ii^	0.98	2.90	3.780 (5)	150
C45—H45*B*⋯O12	0.98	2.46	3.312 (5)	146
N6—H6*N*⋯O9^iii^	0.90 (2)	2.04 (2)	2.933 (4)	172 (5)
C47—H47⋯O11	0.95	2.32	2.899 (6)	118
C51—H51⋯O9^iii^	0.95	2.63	3.409 (5)	139
C53—H53*B*⋯Cl12^iii^	0.98	2.89	3.829 (5)	161
C53—H53*C*⋯O11^iv^	0.98	2.71	3.291 (5)	119
C54—H54*B*⋯O10^v^	0.98	2.59	3.443 (6)	145
N4—H4*N*⋯O3^vi^	0.90 (2)	2.03 (2)	2.924 (4)	170 (5)
C29—H29⋯O7	0.95	2.34	2.915 (6)	118
C33—H33⋯O3^vi^	0.95	2.61	3.389 (5)	140
C35—H35*B*⋯O1^vii^	0.98	2.57	3.311 (5)	133
C36—H36*C*⋯O6^vi^	0.98	2.51	3.438 (6)	157
N3—H3*N*⋯O1^viii^	0.89 (2)	2.08 (2)	2.910 (4)	155 (4)
C20—H20⋯O5	0.95	2.26	2.862 (6)	120
C26—H26*A*⋯O1^viii^	0.98	2.51	3.393 (5)	150
C26—H26*B*⋯Cl4	0.98	2.87	3.771 (5)	154
C26—H26*C*⋯O7	0.98	2.57	3.291 (6)	131
C27—H27*A*⋯Cl10	0.98	2.97	3.604 (5)	123
C27—H27*C*⋯O8	0.98	2.39	3.304 (6)	154
N2—H2*N*⋯O7^ix^	0.89 (2)	2.02 (2)	2.900 (4)	169 (4)
C11—H11⋯O3	0.95	2.24	2.860 (6)	122
C17—H17*A*⋯O7^ix^	0.98	2.56	3.391 (5)	143
C17—H17*C*⋯Cl6	0.98	2.83	3.772 (5)	162
C18—H18*B*⋯O2^v^	0.98	2.47	3.319 (6)	144

Symmetry codes: (i) 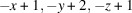; (ii) 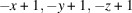; (iii) 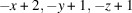; (iv) 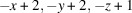; (v) 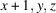; (vi) 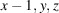; (vii) 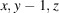; (viii) 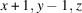; (ix) 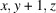.

**Table 2:** Experimental details

Crystal data	
Chemical formula	C_9_H_9_Cl_2_NO_2_
*M*_r_	234.07
Crystal system, space group	Triclinic, *P* 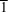
Temperature (K)	100
*a*, *b*, *c* (Å)	8.1860 (8), 8.7093 (8), 44.343 (4)
α, β, γ (°)	84.465 (4), 88.209 (4), 71.399 (3)
*V* (Å^3^)	2982.3 (5)
*Z*	12
Radiation type	Cu *K*α
μ (mm^−1^)	5.66
Crystal size (mm)	0.12 × 0.11 × 0.02

Data collection	
Diffractometer	Bruker D8 Venture DUO with Photon III C14
Absorption correction	Multi-scan (*SADABS*; Krause *et al.*, 2015 ▸)
*T*_min_, *T*_max_	0.679, 0.895
No. of measured, independent and observed [*I* > 2σ(*I*)] reflections	100590, 12442, 6847
*R*_int_	0.067
(sin θ/λ)_max_ (Å^−1^)	0.637

Refinement	
*R*[*F*^2^ > 2σ(*F*^2^)], *wR*(*F*^2^), *S*	0.033, 0.099, 1.03
No. of reflections	12442
No. of parameters	789
No. of restraints	15
H-atom treatment	H atoms treated by a mixture of independent and constrained refinement
Δρ_max_, Δρ_min_ (e Å^−3^)	0.44, −0.50

Computer programs: *APEX5* and *SAINT* (Bruker, 2016 ▸), *SHELXT2018/2* (Sheldrick, 2015*a* ▸), *SHELXL2019/1* (Sheldrick, 2015*b* ▸), *Mercury* (Macrae *et al.*, 2020 ▸) and *publCIF* (Westrip, 2010 ▸).

## full crystallographic data

### N-(3,5-Dichloro-4-methoxyphenyl)acetamide Crystal data

**Table:**

C_9_H_9_Cl_2_NO_2_	*Z* = 12
*M**_r_* = 234.07	*F*(000) = 1440
Triclinic, *P*1	*D*_x_ = 1.564 Mg m^−^^3^
*a* = 8.1860 (8) Å	Cu *K*α radiation, λ = 1.54184 Å
*b* = 8.7093 (8) Å	Cell parameters from 9974 reflections
*c* = 44.343 (4) Å	θ = 5.4–78.4°
α = 84.465 (4)°	µ = 5.66 mm^−^^1^
β = 88.209 (4)°	*T* = 100 K
γ = 71.399 (3)°	Lath fragment, colourless
*V* = 2982.3 (5) Å^3^	0.12 × 0.11 × 0.02 mm

### N-(3,5-Dichloro-4-methoxyphenyl)acetamide Data collection

**Table:**

Bruker D8 Venture DUO with Photon III C14 diffractometer	6847 reflections with *I* > 2σ(*I*)
Radiation source: IµS 3.0 microfocus	*R*_int_ = 0.067
φ and ω scans	θ_max_ = 79.1°, θ_min_ = 2.0°
Absorption correction: multi-scan (SADABS; Krause *et al.*, 2015)	*h* = −10→10
*T*_min_ = 0.679, *T*_max_ = 0.895	*k* = −11→10
100590 measured reflections	*l* = −55→55
12442 independent reflections	

### N-(3,5-Dichloro-4-methoxyphenyl)acetamide Refinement

**Table:**

Refinement on *F*^2^	Hydrogen site location: mixed
Least-squares matrix: full	H atoms treated by a mixture of independent and constrained refinement
*R*[*F*^2^ > 2σ(*F*^2^)] = 0.033	*w* = 1/[σ^2^(*F*_o_^2^) + (0.0445*P*)^2^ + 0.6459*P*] where *P* = (*F*_o_^2^ + 2*F*_c_^2^)/3
*wR*(*F*^2^) = 0.099	(Δ/σ)_max_ = 0.001
*S* = 1.03	Δρ_max_ = 0.44 e Å^−^^3^
12442 reflections	Δρ_min_ = −0.50 e Å^−^^3^
789 parameters	Extinction correction: SHELXL2019/1 (Sheldrick 2015b), Fc^*^=kFc[1+0.001xFc^2^λ^3^/sin(2θ)]^-1/4^
15 restraints	Extinction coefficient: 0.00015 (3)
Primary atom site location: dual	

### N-(3,5-Dichloro-4-methoxyphenyl)acetamide Special details

**Table:**

Geometry. All esds (except the esd in the dihedral angle between two l.s. planes) are estimated using the full covariance matrix. The cell esds are taken into account individually in the estimation of esds in distances, angles and torsion angles; correlations between esds in cell parameters are only used when they are defined by crystal symmetry. An approximate (isotropic) treatment of cell esds is used for estimating esds involving l.s. planes.
Refinement. Refined as a 2-component twin.

### N-(3,5-Dichloro-4-methoxyphenyl)acetamide Fractional atomic coordinates and isotropic or equivalent isotropic displacement parameters (Å^2^)

**Table:**

	*x*	*y*	*z*	*U*_iso_*/*U*_eq_	
Cl1	−0.00483 (15)	0.97432 (12)	0.05545 (3)	0.0268 (2)	
Cl2	0.47196 (16)	0.37931 (11)	0.05965 (3)	0.0255 (2)	
O1	0.1580 (4)	0.9282 (3)	0.16586 (7)	0.0213 (6)	
O2	0.1882 (4)	0.6586 (4)	0.03202 (8)	0.0246 (7)	
N1	0.3698 (5)	0.7079 (4)	0.14998 (9)	0.0187 (7)	
H1N	0.475 (3)	0.635 (4)	0.1534 (11)	0.022*	
C1	0.3177 (6)	0.7077 (5)	0.11994 (10)	0.0184 (8)	
C2	0.1895 (6)	0.8328 (5)	0.10488 (10)	0.0189 (8)	
H2	0.128577	0.927058	0.114605	0.023*	
C3	0.1510 (5)	0.8175 (4)	0.07479 (11)	0.0182 (8)	
C4	0.2366 (6)	0.6783 (5)	0.05979 (11)	0.0208 (9)	
C5	0.3642 (6)	0.5572 (5)	0.07631 (10)	0.0177 (8)	
C6	0.4053 (6)	0.5690 (5)	0.10559 (11)	0.0200 (9)	
H6	0.493070	0.483002	0.116039	0.024*	
C7	0.2925 (6)	0.8119 (5)	0.17087 (10)	0.0180 (8)	
C8	0.3809 (6)	0.7742 (5)	0.20090 (11)	0.0223 (9)	
H8A	0.480478	0.675313	0.200479	0.033*	
H8B	0.300413	0.757218	0.216703	0.033*	
H8C	0.419818	0.865149	0.205219	0.033*	
C9	0.2775 (7)	0.7144 (6)	0.00677 (11)	0.0311 (11)	
H9A	0.246659	0.679406	−0.011999	0.047*	
H9B	0.402217	0.668190	0.009983	0.047*	
H9C	0.244093	0.833418	0.005218	0.047*	
Cl9	0.14784 (13)	0.98252 (11)	0.37794 (3)	0.0224 (2)	
Cl10	0.63149 (13)	0.39432 (10)	0.39756 (3)	0.0201 (2)	
O9	0.6742 (4)	0.5843 (3)	0.49870 (8)	0.0208 (7)	
O10	0.3609 (4)	0.6548 (3)	0.36213 (7)	0.0179 (6)	
N5	0.4396 (4)	0.7919 (3)	0.48021 (9)	0.0159 (7)	
H5N	0.361 (5)	0.882 (4)	0.4858 (12)	0.019*	
C37	0.4303 (5)	0.7519 (4)	0.45073 (10)	0.0122 (7)	
C38	0.5306 (5)	0.6050 (4)	0.43947 (10)	0.0150 (8)	
H38	0.612871	0.524658	0.452060	0.018*	
C39	0.5071 (5)	0.5792 (4)	0.40962 (10)	0.0148 (8)	
C40	0.3910 (5)	0.6900 (4)	0.39042 (11)	0.0155 (8)	
C41	0.2938 (5)	0.8361 (4)	0.40140 (11)	0.0165 (8)	
C42	0.3123 (5)	0.8687 (4)	0.43071 (10)	0.0147 (8)	
H42	0.245419	0.970038	0.437520	0.018*	
C43	0.5556 (5)	0.7111 (4)	0.50249 (11)	0.0170 (8)	
C44	0.5286 (5)	0.7891 (4)	0.53143 (11)	0.0173 (8)	
H44A	0.479197	0.907305	0.527169	0.026*	
H44B	0.639311	0.763272	0.541827	0.026*	
H44C	0.449483	0.748193	0.544375	0.026*	
C45	0.4658 (6)	0.7017 (5)	0.33853 (11)	0.0216 (9)	
H45A	0.435942	0.673462	0.319028	0.032*	
H45B	0.587735	0.644149	0.342938	0.032*	
H45C	0.444777	0.819290	0.337605	0.032*	
Cl11	0.66132 (13)	0.97484 (11)	0.38871 (3)	0.0228 (2)	
Cl12	1.13883 (14)	0.37932 (10)	0.39285 (3)	0.0219 (2)	
O11	0.8284 (4)	0.9267 (3)	0.49911 (8)	0.0187 (6)	
O12	0.8529 (4)	0.6592 (3)	0.36509 (8)	0.0196 (6)	
N6	1.0365 (4)	0.7074 (3)	0.48312 (9)	0.0162 (7)	
H6N	1.124 (5)	0.621 (4)	0.4906 (12)	0.019*	
C46	0.9859 (5)	0.7058 (4)	0.45352 (10)	0.0148 (8)	
C47	0.8566 (5)	0.8326 (4)	0.43774 (11)	0.0168 (8)	
H47	0.796216	0.927886	0.447207	0.020*	
C48	0.8185 (5)	0.8165 (4)	0.40814 (11)	0.0174 (8)	
C49	0.9033 (5)	0.6776 (4)	0.39348 (10)	0.0152 (8)	
C50	1.0322 (5)	0.5558 (4)	0.40942 (11)	0.0167 (8)	
C51	1.0733 (5)	0.5683 (4)	0.43877 (11)	0.0156 (8)	
H51	1.161907	0.482496	0.449082	0.019*	
C52	0.9593 (5)	0.8102 (4)	0.50426 (10)	0.0159 (8)	
C53	1.0489 (5)	0.7750 (4)	0.53471 (11)	0.0202 (9)	
H53A	1.164119	0.696101	0.532945	0.030*	
H53B	0.981555	0.730051	0.549656	0.030*	
H53C	1.058889	0.875932	0.541243	0.030*	
C54	0.9436 (6)	0.7131 (5)	0.34042 (12)	0.0274 (10)	
H54A	0.900187	0.694115	0.321245	0.041*	
H54B	1.066935	0.652631	0.342248	0.041*	
H54C	0.926054	0.829582	0.340847	0.041*	
Cl7	0.67080 (14)	0.02552 (11)	0.27795 (3)	0.0242 (2)	
Cl8	0.19360 (15)	0.62021 (11)	0.27378 (3)	0.0230 (2)	
O7	0.5051 (4)	0.0727 (3)	0.16761 (7)	0.0201 (6)	
O8	0.4796 (4)	0.3422 (3)	0.30151 (8)	0.0192 (6)	
N4	0.2948 (5)	0.2923 (4)	0.18317 (9)	0.0169 (7)	
H4N	0.211 (5)	0.382 (4)	0.1755 (12)	0.020*	
C28	0.3486 (5)	0.2934 (4)	0.21338 (10)	0.0159 (8)	
C29	0.4759 (5)	0.1688 (4)	0.22922 (11)	0.0186 (9)	
H29	0.537412	0.073352	0.219889	0.022*	
C30	0.5112 (5)	0.1857 (4)	0.25838 (11)	0.0182 (8)	
C31	0.4322 (6)	0.3209 (4)	0.27336 (11)	0.0178 (9)	
C32	0.3037 (6)	0.4447 (4)	0.25708 (11)	0.0184 (9)	
C33	0.2582 (5)	0.4317 (4)	0.22761 (10)	0.0173 (8)	
H33	0.167199	0.515508	0.217413	0.021*	
C34	0.3722 (5)	0.1887 (4)	0.16262 (10)	0.0165 (8)	
C35	0.2848 (6)	0.2272 (4)	0.13218 (10)	0.0203 (9)	
H35A	0.221669	0.343850	0.129172	0.030*	
H35B	0.204084	0.165305	0.131346	0.030*	
H35C	0.371634	0.197471	0.116217	0.030*	
C36	0.3881 (6)	0.2836 (6)	0.32625 (11)	0.0245 (9)	
H36A	0.417588	0.316102	0.345373	0.037*	
H36B	0.421315	0.164698	0.327270	0.037*	
H36C	0.263635	0.330427	0.322790	0.037*	
Cl5	1.18368 (14)	0.01843 (11)	0.28875 (3)	0.0237 (2)	
Cl6	0.70077 (14)	0.60680 (11)	0.26920 (3)	0.0222 (2)	
O5	0.6582 (4)	0.4151 (3)	0.16794 (8)	0.0231 (7)	
O6	0.9707 (4)	0.3466 (3)	0.30442 (8)	0.0198 (6)	
N3	0.8929 (5)	0.2097 (4)	0.18647 (9)	0.0184 (7)	
H3N	0.953 (5)	0.108 (3)	0.1830 (11)	0.022*	
C19	0.9015 (5)	0.2478 (4)	0.21581 (11)	0.0195 (9)	
C20	0.8034 (5)	0.3936 (5)	0.22667 (11)	0.0196 (9)	
H20	0.722681	0.473728	0.213786	0.024*	
C21	0.8234 (5)	0.4217 (4)	0.25607 (12)	0.0194 (9)	
C22	0.9444 (5)	0.3112 (4)	0.27637 (10)	0.0165 (9)	
C23	1.0402 (5)	0.1646 (4)	0.26459 (11)	0.0191 (9)	
C24	1.0205 (6)	0.1348 (4)	0.23504 (11)	0.0191 (9)	
H24	1.089688	0.035370	0.227819	0.023*	
C25	0.7743 (6)	0.2903 (5)	0.16449 (11)	0.0189 (9)	
C26	0.8020 (6)	0.2120 (4)	0.13468 (11)	0.0233 (9)	
H26A	0.907911	0.118589	0.135892	0.035*	
H26B	0.812176	0.291635	0.118147	0.035*	
H26C	0.703834	0.175306	0.130819	0.035*	
C27	0.8663 (6)	0.2972 (5)	0.32811 (11)	0.0229 (9)	
H27A	0.886784	0.335289	0.347283	0.034*	
H27B	0.897545	0.178263	0.330323	0.034*	
H27C	0.744171	0.344591	0.322689	0.034*	
Cl3	0.48165 (15)	0.98228 (12)	0.04455 (3)	0.0257 (2)	
Cl4	0.96522 (15)	0.39414 (11)	0.06414 (3)	0.0238 (2)	
O3	1.0073 (4)	0.5849 (3)	0.16548 (8)	0.0240 (7)	
O4	0.6939 (4)	0.6544 (4)	0.02870 (7)	0.0229 (7)	
N2	0.7712 (5)	0.7930 (4)	0.14728 (9)	0.0166 (7)	
H2N	0.679 (4)	0.871 (4)	0.1534 (11)	0.020*	
C10	0.7613 (6)	0.7527 (5)	0.11650 (10)	0.0175 (8)	
C11	0.8634 (6)	0.6067 (5)	0.10666 (11)	0.0197 (9)	
H11	0.946091	0.529510	0.119663	0.024*	
C12	0.8408 (6)	0.5766 (5)	0.07694 (10)	0.0185 (8)	
C13	0.7234 (6)	0.6884 (5)	0.05729 (11)	0.0207 (9)	
C14	0.6279 (6)	0.8349 (4)	0.06840 (10)	0.0180 (9)	
C15	0.6435 (6)	0.8686 (5)	0.09760 (10)	0.0196 (8)	
H15	0.575325	0.968817	0.104654	0.023*	
C16	0.8878 (6)	0.7122 (4)	0.16864 (10)	0.0156 (8)	
C17	0.8620 (5)	0.7887 (5)	0.19837 (10)	0.0187 (8)	
H17A	0.781361	0.899664	0.195428	0.028*	
H17B	0.972804	0.791930	0.205511	0.028*	
H17C	0.814938	0.724096	0.213432	0.028*	
C18	0.7998 (6)	0.7026 (6)	0.00546 (11)	0.0274 (10)	
H18A	0.768863	0.678187	−0.014291	0.041*	
H18B	0.921413	0.642679	0.009711	0.041*	
H18C	0.780983	0.819614	0.005182	0.041*	

### N-(3,5-Dichloro-4-methoxyphenyl)acetamide Atomic displacement parameters (Å^2^)

**Table:**

	*U*^11^	*U*^22^	*U*^33^	*U*^12^	*U*^13^	*U*^23^
Cl1	0.0279 (5)	0.0257 (4)	0.0232 (6)	−0.0036 (4)	−0.0078 (5)	−0.0002 (4)
Cl2	0.0362 (6)	0.0218 (4)	0.0170 (6)	−0.0053 (4)	0.0024 (5)	−0.0092 (4)
O1	0.0229 (15)	0.0217 (12)	0.0161 (17)	−0.0021 (11)	−0.0024 (13)	−0.0032 (11)
O2	0.0284 (16)	0.0358 (15)	0.0135 (17)	−0.0147 (13)	−0.0009 (14)	−0.0056 (12)
N1	0.0183 (16)	0.0195 (15)	0.0169 (19)	−0.0036 (12)	0.0021 (15)	−0.0048 (13)
C1	0.0210 (19)	0.0225 (18)	0.015 (2)	−0.0128 (15)	0.0048 (18)	−0.0022 (15)
C2	0.026 (2)	0.0221 (17)	0.011 (2)	−0.0093 (15)	−0.0040 (17)	−0.0052 (14)
C3	0.0176 (18)	0.0199 (17)	0.016 (2)	−0.0048 (14)	0.0006 (17)	0.0012 (15)
C4	0.026 (2)	0.0237 (18)	0.016 (2)	−0.0134 (16)	0.006 (2)	0.0021 (16)
C5	0.022 (2)	0.0201 (17)	0.012 (2)	−0.0066 (15)	0.0039 (18)	−0.0060 (15)
C6	0.027 (2)	0.0166 (16)	0.018 (2)	−0.0089 (15)	0.0017 (19)	−0.0023 (15)
C7	0.024 (2)	0.0198 (17)	0.014 (2)	−0.0110 (15)	0.0057 (18)	−0.0051 (14)
C8	0.024 (2)	0.0242 (18)	0.018 (2)	−0.0070 (15)	0.0049 (19)	−0.0008 (16)
C9	0.037 (3)	0.042 (2)	0.012 (2)	−0.010 (2)	−0.003 (2)	0.0008 (18)
Cl9	0.0207 (5)	0.0161 (4)	0.0273 (6)	−0.0012 (3)	−0.0087 (4)	−0.0006 (4)
Cl10	0.0210 (5)	0.0142 (4)	0.0221 (5)	0.0000 (3)	0.0014 (4)	−0.0070 (3)
O9	0.0182 (14)	0.0112 (11)	0.0266 (18)	0.0048 (10)	−0.0048 (13)	−0.0027 (11)
O10	0.0218 (14)	0.0219 (12)	0.0145 (16)	−0.0123 (10)	0.0005 (13)	−0.0043 (11)
N5	0.0131 (15)	0.0113 (13)	0.0188 (19)	0.0036 (11)	−0.0044 (15)	−0.0038 (12)
C37	0.0121 (17)	0.0123 (15)	0.012 (2)	−0.0034 (13)	−0.0023 (16)	−0.0005 (13)
C38	0.0099 (15)	0.0057 (14)	0.024 (2)	0.0046 (11)	0.0014 (16)	−0.0002 (13)
C39	0.0138 (17)	0.0135 (15)	0.020 (2)	−0.0057 (13)	0.0086 (16)	−0.0147 (14)
C40	0.0146 (18)	0.0171 (16)	0.017 (2)	−0.0066 (13)	0.0007 (17)	−0.0052 (14)
C41	0.0140 (17)	0.0129 (15)	0.024 (2)	−0.0057 (13)	0.0014 (18)	−0.0029 (14)
C42	0.0093 (16)	0.0075 (14)	0.026 (2)	0.0012 (12)	−0.0008 (17)	−0.0050 (14)
C43	0.0093 (17)	0.0127 (16)	0.027 (2)	−0.0007 (12)	−0.0038 (17)	0.0018 (15)
C44	0.0138 (17)	0.0175 (16)	0.021 (2)	−0.0052 (13)	0.0011 (17)	−0.0013 (14)
C45	0.022 (2)	0.0288 (19)	0.016 (2)	−0.0115 (15)	−0.0027 (18)	0.0021 (16)
Cl11	0.0167 (4)	0.0178 (4)	0.0299 (6)	0.0002 (3)	−0.0082 (4)	0.0000 (4)
Cl12	0.0236 (5)	0.0155 (4)	0.0238 (6)	−0.0008 (3)	0.0005 (4)	−0.0076 (4)
O11	0.0147 (13)	0.0116 (11)	0.0264 (17)	0.0010 (9)	0.0029 (13)	−0.0048 (11)
O12	0.0185 (14)	0.0260 (13)	0.0187 (17)	−0.0119 (10)	−0.0027 (13)	−0.0062 (12)
N6	0.0136 (15)	0.0116 (13)	0.022 (2)	−0.0012 (11)	−0.0030 (15)	−0.0036 (12)
C46	0.0123 (17)	0.0128 (15)	0.018 (2)	−0.0034 (13)	0.0018 (17)	0.0015 (14)
C47	0.0128 (17)	0.0109 (15)	0.027 (2)	−0.0028 (13)	0.0037 (18)	−0.0065 (14)
C48	0.0154 (18)	0.0159 (16)	0.021 (2)	−0.0055 (14)	−0.0032 (18)	0.0010 (15)
C49	0.0116 (17)	0.0222 (16)	0.016 (2)	−0.0094 (13)	0.0001 (17)	−0.0099 (15)
C50	0.0108 (17)	0.0163 (16)	0.023 (2)	−0.0038 (13)	0.0005 (17)	−0.0016 (15)
C51	0.0094 (16)	0.0122 (15)	0.023 (2)	−0.0002 (12)	0.0007 (16)	0.0003 (14)
C52	0.0164 (18)	0.0150 (15)	0.019 (2)	−0.0077 (13)	0.0021 (17)	−0.0038 (14)
C53	0.0190 (19)	0.0152 (16)	0.026 (3)	−0.0024 (14)	−0.0049 (18)	−0.0075 (15)
C54	0.028 (2)	0.036 (2)	0.024 (3)	−0.0177 (18)	−0.002 (2)	−0.0055 (18)
Cl7	0.0222 (5)	0.0224 (4)	0.0245 (6)	−0.0022 (3)	−0.0059 (5)	−0.0002 (4)
Cl8	0.0274 (5)	0.0204 (4)	0.0192 (5)	−0.0035 (4)	0.0008 (5)	−0.0066 (4)
O7	0.0224 (15)	0.0186 (12)	0.0172 (16)	−0.0031 (10)	0.0027 (13)	−0.0046 (11)
O8	0.0211 (15)	0.0247 (13)	0.0144 (16)	−0.0108 (11)	−0.0045 (13)	−0.0001 (11)
N4	0.0165 (15)	0.0191 (14)	0.0113 (18)	−0.0007 (12)	−0.0029 (14)	−0.0001 (12)
C28	0.0169 (18)	0.0190 (17)	0.015 (2)	−0.0075 (14)	−0.0011 (17)	−0.0092 (14)
C29	0.0120 (17)	0.0171 (17)	0.024 (2)	−0.0020 (13)	0.0023 (18)	0.0006 (15)
C30	0.0155 (18)	0.0174 (17)	0.025 (2)	−0.0084 (13)	−0.0039 (18)	−0.0043 (15)
C31	0.019 (2)	0.0195 (18)	0.016 (2)	−0.0079 (15)	−0.0039 (18)	0.0006 (15)
C32	0.022 (2)	0.0152 (16)	0.020 (2)	−0.0082 (14)	0.0042 (19)	−0.0072 (15)
C33	0.0169 (18)	0.0199 (16)	0.014 (2)	−0.0037 (14)	−0.0035 (17)	−0.0038 (15)
C34	0.0171 (18)	0.0126 (15)	0.019 (2)	−0.0034 (13)	−0.0012 (18)	0.0004 (14)
C35	0.029 (2)	0.0194 (17)	0.010 (2)	−0.0046 (15)	−0.0044 (18)	0.0001 (14)
C36	0.026 (2)	0.041 (2)	0.013 (2)	−0.0201 (18)	0.0015 (19)	−0.0015 (17)
Cl5	0.0258 (5)	0.0186 (4)	0.0227 (6)	−0.0012 (3)	−0.0089 (5)	−0.0005 (4)
Cl6	0.0263 (5)	0.0169 (4)	0.0194 (5)	−0.0003 (4)	0.0014 (4)	−0.0050 (4)
O5	0.0181 (15)	0.0247 (14)	0.0210 (18)	0.0019 (11)	−0.0035 (14)	−0.0052 (12)
O6	0.0164 (14)	0.0222 (12)	0.0224 (17)	−0.0068 (10)	−0.0013 (13)	−0.0076 (11)
N3	0.0194 (16)	0.0172 (14)	0.018 (2)	−0.0052 (12)	0.0025 (15)	−0.0040 (13)
C19	0.0163 (18)	0.0125 (16)	0.029 (3)	−0.0048 (13)	0.0002 (18)	0.0010 (15)
C20	0.020 (2)	0.0216 (18)	0.016 (2)	−0.0064 (15)	−0.0024 (18)	0.0019 (15)
C21	0.0161 (18)	0.0126 (16)	0.028 (3)	−0.0028 (13)	−0.0006 (19)	0.0007 (15)
C22	0.020 (2)	0.0189 (18)	0.014 (2)	−0.0116 (15)	0.0001 (18)	0.0008 (15)
C23	0.0153 (18)	0.0173 (16)	0.025 (2)	−0.0052 (14)	−0.0014 (18)	−0.0034 (15)
C24	0.0203 (19)	0.0150 (16)	0.023 (2)	−0.0060 (14)	−0.0003 (19)	−0.0032 (15)
C25	0.022 (2)	0.0194 (17)	0.018 (2)	−0.0107 (15)	0.0016 (18)	0.0004 (15)
C26	0.032 (2)	0.0137 (16)	0.021 (2)	−0.0026 (15)	−0.002 (2)	−0.0017 (15)
C27	0.023 (2)	0.0290 (19)	0.017 (2)	−0.0089 (16)	0.0010 (18)	−0.0025 (16)
Cl3	0.0314 (6)	0.0228 (4)	0.0197 (6)	−0.0035 (4)	−0.0083 (5)	−0.0007 (4)
Cl4	0.0329 (5)	0.0195 (4)	0.0153 (5)	−0.0024 (4)	0.0034 (4)	−0.0046 (3)
O3	0.0274 (17)	0.0233 (14)	0.0177 (17)	−0.0035 (12)	0.0027 (14)	−0.0009 (12)
O4	0.0304 (16)	0.0269 (14)	0.0128 (16)	−0.0104 (12)	−0.0027 (14)	−0.0028 (11)
N2	0.0212 (16)	0.0158 (14)	0.0116 (18)	−0.0036 (12)	0.0022 (15)	−0.0036 (12)
C10	0.0216 (19)	0.0288 (18)	0.0062 (19)	−0.0131 (15)	0.0060 (17)	−0.0066 (15)
C11	0.027 (2)	0.0235 (18)	0.012 (2)	−0.0109 (16)	0.0007 (18)	−0.0067 (15)
C12	0.024 (2)	0.0200 (17)	0.011 (2)	−0.0076 (14)	−0.0007 (17)	0.0044 (14)
C13	0.021 (2)	0.0270 (19)	0.017 (2)	−0.0120 (16)	0.0003 (19)	−0.0041 (16)
C14	0.023 (2)	0.0190 (17)	0.011 (2)	−0.0061 (14)	−0.0053 (17)	0.0049 (14)
C15	0.024 (2)	0.0223 (17)	0.012 (2)	−0.0066 (15)	0.0030 (18)	−0.0002 (15)
C16	0.0216 (19)	0.0163 (16)	0.009 (2)	−0.0056 (14)	0.0057 (16)	−0.0057 (13)
C17	0.0183 (19)	0.0306 (19)	0.007 (2)	−0.0065 (15)	−0.0039 (17)	−0.0047 (15)
C18	0.026 (2)	0.043 (2)	0.012 (2)	−0.0094 (18)	−0.0020 (19)	−0.0036 (18)

### N-(3,5-Dichloro-4-methoxyphenyl)acetamide Geometric parameters (Å, º)

**Table:**

Cl1—C3	1.720 (4)	Cl7—C30	1.755 (4)
Cl2—C5	1.741 (4)	Cl8—C32	1.731 (4)
O1—C7	1.244 (5)	O7—C34	1.234 (5)
O2—C4	1.348 (6)	O8—C31	1.365 (6)
O2—C9	1.447 (6)	O8—C36	1.453 (6)
N1—C7	1.356 (5)	N4—C34	1.343 (5)
N1—C1	1.412 (6)	N4—C28	1.425 (6)
N1—H1N	0.899 (17)	N4—H4N	0.901 (17)
C1—C2	1.382 (6)	C28—C29	1.391 (5)
C1—C6	1.395 (6)	C28—C33	1.395 (5)
C2—C3	1.409 (6)	C29—C30	1.365 (7)
C2—H2	0.9500	C29—H29	0.9500
C3—C4	1.410 (6)	C30—C31	1.373 (6)
C4—C5	1.392 (6)	C31—C32	1.402 (6)
C5—C6	1.373 (7)	C32—C33	1.395 (7)
C6—H6	0.9500	C33—H33	0.9500
C7—C8	1.493 (7)	C34—C35	1.505 (6)
C8—H8A	0.9800	C35—H35A	0.9800
C8—H8B	0.9800	C35—H35B	0.9800
C8—H8C	0.9800	C35—H35C	0.9800
C9—H9A	0.9800	C36—H36A	0.9800
C9—H9B	0.9800	C36—H36B	0.9800
C9—H9C	0.9800	C36—H36C	0.9800
Cl9—C41	1.728 (4)	Cl5—C23	1.735 (4)
Cl10—C39	1.733 (3)	Cl6—C21	1.749 (4)
O9—C43	1.237 (5)	O5—C25	1.213 (5)
O10—C40	1.367 (6)	O6—C22	1.350 (6)
O10—C45	1.443 (6)	O6—C27	1.453 (6)
N5—C43	1.368 (5)	N3—C25	1.370 (6)
N5—C37	1.395 (6)	N3—C19	1.383 (6)
N5—H5N	0.895 (17)	N3—H3N	0.891 (17)
C37—C38	1.410 (5)	C19—C24	1.387 (6)
C37—C42	1.417 (5)	C19—C20	1.390 (6)
C38—C39	1.393 (6)	C20—C21	1.373 (7)
C38—H38	0.9500	C20—H20	0.9500
C39—C40	1.366 (6)	C21—C22	1.413 (6)
C40—C41	1.392 (5)	C22—C23	1.408 (6)
C41—C42	1.380 (7)	C23—C24	1.384 (7)
C42—H42	0.9500	C24—H24	0.9500
C43—C44	1.487 (6)	C25—C26	1.524 (6)
C44—H44A	0.9800	C26—H26A	0.9800
C44—H44B	0.9800	C26—H26B	0.9800
C44—H44C	0.9800	C26—H26C	0.9800
C45—H45A	0.9800	C27—H27A	0.9800
C45—H45B	0.9800	C27—H27B	0.9800
C45—H45C	0.9800	C27—H27C	0.9800
Cl11—C48	1.735 (4)	Cl3—C14	1.742 (4)
Cl12—C50	1.728 (4)	Cl4—C12	1.729 (4)
O11—C52	1.228 (5)	O3—C16	1.239 (5)
O12—C49	1.376 (6)	O4—C13	1.375 (6)
O12—C54	1.429 (6)	O4—C18	1.443 (6)
N6—C52	1.358 (6)	N2—C16	1.343 (6)
N6—C46	1.391 (6)	N2—C10	1.453 (5)
N6—H6N	0.902 (17)	N2—H2N	0.894 (17)
C46—C51	1.396 (5)	C10—C11	1.382 (6)
C46—C47	1.407 (5)	C10—C15	1.384 (6)
C47—C48	1.389 (7)	C11—C12	1.397 (7)
C47—H47	0.9500	C11—H11	0.9500
C48—C49	1.398 (6)	C12—C13	1.387 (6)
C49—C50	1.388 (5)	C13—C14	1.394 (6)
C50—C51	1.377 (7)	C14—C15	1.374 (6)
C51—H51	0.9500	C15—H15	0.9500
C52—C53	1.514 (6)	C16—C17	1.513 (6)
C53—H53A	0.9800	C17—H17A	0.9800
C53—H53B	0.9800	C17—H17B	0.9800
C53—H53C	0.9800	C17—H17C	0.9800
C54—H54A	0.9800	C18—H18A	0.9800
C54—H54B	0.9800	C18—H18B	0.9800
C54—H54C	0.9800	C18—H18C	0.9800
			
C4—O2—C9	115.9 (4)	C31—O8—C36	114.4 (3)
C7—N1—C1	128.5 (4)	C34—N4—C28	128.0 (3)
C7—N1—H1N	121 (3)	C34—N4—H4N	114 (4)
C1—N1—H1N	110 (3)	C28—N4—H4N	117 (4)
C2—C1—C6	120.3 (4)	C29—C28—C33	119.6 (4)
C2—C1—N1	124.4 (4)	C29—C28—N4	125.6 (4)
C6—C1—N1	115.3 (4)	C33—C28—N4	114.7 (4)
C1—C2—C3	118.6 (4)	C30—C29—C28	119.1 (4)
C1—C2—H2	120.7	C30—C29—H29	120.5
C3—C2—H2	120.7	C28—C29—H29	120.5
C2—C3—C4	122.3 (4)	C29—C30—C31	124.4 (4)
C2—C3—Cl1	119.1 (3)	C29—C30—Cl7	118.1 (3)
C4—C3—Cl1	118.6 (4)	C31—C30—Cl7	117.5 (4)
O2—C4—C5	122.0 (4)	O8—C31—C30	123.7 (4)
O2—C4—C3	121.8 (4)	O8—C31—C32	120.5 (4)
C5—C4—C3	116.0 (4)	C30—C31—C32	115.6 (4)
C6—C5—C4	123.0 (4)	C33—C32—C31	122.4 (4)
C6—C5—Cl2	118.3 (3)	C33—C32—Cl8	117.9 (3)
C4—C5—Cl2	118.7 (3)	C31—C32—Cl8	119.8 (4)
C5—C6—C1	119.8 (4)	C32—C33—C28	118.8 (4)
C5—C6—H6	120.1	C32—C33—H33	120.6
C1—C6—H6	120.1	C28—C33—H33	120.6
O1—C7—N1	123.4 (4)	O7—C34—N4	123.9 (4)
O1—C7—C8	122.2 (4)	O7—C34—C35	121.9 (4)
N1—C7—C8	114.3 (4)	N4—C34—C35	114.2 (3)
C7—C8—H8A	109.5	C34—C35—H35A	109.5
C7—C8—H8B	109.5	C34—C35—H35B	109.5
H8A—C8—H8B	109.5	H35A—C35—H35B	109.5
C7—C8—H8C	109.5	C34—C35—H35C	109.5
H8A—C8—H8C	109.5	H35A—C35—H35C	109.5
H8B—C8—H8C	109.5	H35B—C35—H35C	109.5
O2—C9—H9A	109.5	O8—C36—H36A	109.5
O2—C9—H9B	109.5	O8—C36—H36B	109.5
H9A—C9—H9B	109.5	H36A—C36—H36B	109.5
O2—C9—H9C	109.5	O8—C36—H36C	109.5
H9A—C9—H9C	109.5	H36A—C36—H36C	109.5
H9B—C9—H9C	109.5	H36B—C36—H36C	109.5
C40—O10—C45	114.4 (3)	C22—O6—C27	115.0 (3)
C43—N5—C37	128.7 (3)	C25—N3—C19	128.3 (4)
C43—N5—H5N	114 (4)	C25—N3—H3N	114 (3)
C37—N5—H5N	118 (4)	C19—N3—H3N	115 (3)
N5—C37—C38	124.8 (3)	N3—C19—C24	117.2 (4)
N5—C37—C42	117.3 (3)	N3—C19—C20	124.2 (4)
C38—C37—C42	118.0 (4)	C24—C19—C20	118.6 (4)
C39—C38—C37	119.0 (3)	C21—C20—C19	119.9 (4)
C39—C38—H38	120.5	C21—C20—H20	120.1
C37—C38—H38	120.5	C19—C20—H20	120.1
C40—C39—C38	123.2 (3)	C20—C21—C22	123.5 (4)
C40—C39—Cl10	120.3 (3)	C20—C21—Cl6	119.2 (3)
C38—C39—Cl10	116.5 (3)	C22—C21—Cl6	117.2 (4)
C39—C40—O10	121.5 (3)	O6—C22—C23	122.7 (4)
C39—C40—C41	117.8 (4)	O6—C22—C21	122.4 (4)
O10—C40—C41	120.6 (4)	C23—C22—C21	114.8 (4)
C42—C41—C40	121.6 (4)	C24—C23—C22	122.1 (4)
C42—C41—Cl9	118.5 (3)	C24—C23—Cl5	120.4 (3)
C40—C41—Cl9	119.9 (4)	C22—C23—Cl5	117.5 (4)
C41—C42—C37	120.4 (3)	C23—C24—C19	121.0 (4)
C41—C42—H42	119.8	C23—C24—H24	119.5
C37—C42—H42	119.8	C19—C24—H24	119.5
O9—C43—N5	122.6 (4)	O5—C25—N3	123.6 (4)
O9—C43—C44	122.9 (4)	O5—C25—C26	122.1 (4)
N5—C43—C44	114.5 (3)	N3—C25—C26	114.3 (4)
C43—C44—H44A	109.5	C25—C26—H26A	109.5
C43—C44—H44B	109.5	C25—C26—H26B	109.5
H44A—C44—H44B	109.5	H26A—C26—H26B	109.5
C43—C44—H44C	109.5	C25—C26—H26C	109.5
H44A—C44—H44C	109.5	H26A—C26—H26C	109.5
H44B—C44—H44C	109.5	H26B—C26—H26C	109.5
O10—C45—H45A	109.5	O6—C27—H27A	109.5
O10—C45—H45B	109.5	O6—C27—H27B	109.5
H45A—C45—H45B	109.5	H27A—C27—H27B	109.5
O10—C45—H45C	109.5	O6—C27—H27C	109.5
H45A—C45—H45C	109.5	H27A—C27—H27C	109.5
H45B—C45—H45C	109.5	H27B—C27—H27C	109.5
C49—O12—C54	115.3 (3)	C13—O4—C18	114.1 (4)
C52—N6—C46	129.3 (3)	C16—N2—C10	127.8 (3)
C52—N6—H6N	113 (4)	C16—N2—H2N	117 (3)
C46—N6—H6N	117 (4)	C10—N2—H2N	115 (3)
N6—C46—C51	116.4 (3)	C11—C10—C15	121.9 (4)
N6—C46—C47	124.7 (4)	C11—C10—N2	121.9 (4)
C51—C46—C47	118.8 (4)	C15—C10—N2	116.2 (4)
C48—C47—C46	119.1 (3)	C10—C11—C12	117.8 (4)
C48—C47—H47	120.4	C10—C11—H11	121.1
C46—C47—H47	120.4	C12—C11—H11	121.1
C47—C48—C49	122.2 (4)	C13—C12—C11	122.3 (4)
C47—C48—Cl11	118.7 (3)	C13—C12—Cl4	118.9 (4)
C49—C48—Cl11	119.0 (4)	C11—C12—Cl4	118.8 (3)
O12—C49—C50	121.6 (4)	O4—C13—C12	122.1 (4)
O12—C49—C48	120.9 (4)	O4—C13—C14	120.8 (4)
C50—C49—C48	117.4 (4)	C12—C13—C14	117.0 (4)
C51—C50—C49	121.8 (4)	C15—C14—C13	122.7 (4)
C51—C50—Cl12	118.6 (3)	C15—C14—Cl3	118.1 (3)
C49—C50—Cl12	119.6 (3)	C13—C14—Cl3	119.2 (4)
C50—C51—C46	120.6 (3)	C14—C15—C10	118.3 (4)
C50—C51—H51	119.7	C14—C15—H15	120.8
C46—C51—H51	119.7	C10—C15—H15	120.8
O11—C52—N6	122.9 (4)	O3—C16—N2	125.1 (4)
O11—C52—C53	121.6 (4)	O3—C16—C17	120.7 (4)
N6—C52—C53	115.4 (3)	N2—C16—C17	114.2 (3)
C52—C53—H53A	109.5	C16—C17—H17A	109.5
C52—C53—H53B	109.5	C16—C17—H17B	109.5
H53A—C53—H53B	109.5	H17A—C17—H17B	109.5
C52—C53—H53C	109.5	C16—C17—H17C	109.5
H53A—C53—H53C	109.5	H17A—C17—H17C	109.5
H53B—C53—H53C	109.5	H17B—C17—H17C	109.5
O12—C54—H54A	109.5	O4—C18—H18A	109.5
O12—C54—H54B	109.5	O4—C18—H18B	109.5
H54A—C54—H54B	109.5	H18A—C18—H18B	109.5
O12—C54—H54C	109.5	O4—C18—H18C	109.5
H54A—C54—H54C	109.5	H18A—C18—H18C	109.5
H54B—C54—H54C	109.5	H18B—C18—H18C	109.5
			
C7—N1—C1—C2	10.9 (7)	C34—N4—C28—C29	−12.4 (7)
C7—N1—C1—C6	−169.0 (4)	C34—N4—C28—C33	169.7 (4)
C6—C1—C2—C3	−0.7 (7)	C33—C28—C29—C30	−0.9 (7)
N1—C1—C2—C3	179.5 (4)	N4—C28—C29—C30	−178.6 (4)
C1—C2—C3—C4	1.1 (7)	C28—C29—C30—C31	−1.8 (7)
C1—C2—C3—Cl1	−178.5 (3)	C28—C29—C30—Cl7	179.3 (3)
C9—O2—C4—C5	−92.2 (5)	C36—O8—C31—C30	−91.2 (5)
C9—O2—C4—C3	93.1 (5)	C36—O8—C31—C32	93.0 (5)
C2—C3—C4—O2	173.9 (4)	C29—C30—C31—O8	−173.8 (4)
Cl1—C3—C4—O2	−6.5 (6)	Cl7—C30—C31—O8	5.0 (6)
C2—C3—C4—C5	−1.1 (7)	C29—C30—C31—C32	2.1 (7)
Cl1—C3—C4—C5	178.5 (3)	Cl7—C30—C31—C32	−179.0 (3)
O2—C4—C5—C6	−174.3 (4)	O8—C31—C32—C33	176.3 (4)
C3—C4—C5—C6	0.7 (7)	C30—C31—C32—C33	0.2 (7)
O2—C4—C5—Cl2	3.4 (6)	O8—C31—C32—Cl8	−4.5 (6)
C3—C4—C5—Cl2	178.4 (3)	C30—C31—C32—Cl8	179.3 (3)
C4—C5—C6—C1	−0.3 (7)	C31—C32—C33—C28	−2.7 (7)
Cl2—C5—C6—C1	−178.0 (3)	Cl8—C32—C33—C28	178.1 (3)
C2—C1—C6—C5	0.3 (7)	C29—C28—C33—C32	3.0 (7)
N1—C1—C6—C5	−179.9 (4)	N4—C28—C33—C32	−179.0 (4)
C1—N1—C7—O1	−0.4 (7)	C28—N4—C34—O7	1.6 (7)
C1—N1—C7—C8	178.7 (4)	C28—N4—C34—C35	−177.2 (4)
C43—N5—C37—C38	8.3 (7)	C25—N3—C19—C24	171.5 (4)
C43—N5—C37—C42	−171.2 (4)	C25—N3—C19—C20	−10.9 (7)
N5—C37—C38—C39	179.2 (4)	N3—C19—C20—C21	−178.7 (4)
C42—C37—C38—C39	−1.3 (6)	C24—C19—C20—C21	−1.2 (6)
C37—C38—C39—C40	0.0 (6)	C19—C20—C21—C22	2.1 (7)
C37—C38—C39—Cl10	−178.8 (3)	C19—C20—C21—Cl6	179.2 (3)
C38—C39—C40—O10	−174.3 (4)	C27—O6—C22—C23	−91.1 (5)
Cl10—C39—C40—O10	4.5 (6)	C27—O6—C22—C21	91.7 (5)
C38—C39—C40—C41	0.9 (6)	C20—C21—C22—O6	174.9 (4)
Cl10—C39—C40—C41	179.6 (3)	Cl6—C21—C22—O6	−2.3 (6)
C45—O10—C40—C39	−92.3 (5)	C20—C21—C22—C23	−2.5 (7)
C45—O10—C40—C41	92.7 (5)	Cl6—C21—C22—C23	−179.7 (3)
C39—C40—C41—C42	−0.4 (6)	O6—C22—C23—C24	−175.2 (4)
O10—C40—C41—C42	174.8 (4)	C21—C22—C23—C24	2.2 (6)
C39—C40—C41—Cl9	178.7 (3)	O6—C22—C23—Cl5	5.8 (6)
O10—C40—C41—Cl9	−6.1 (6)	C21—C22—C23—Cl5	−176.8 (3)
C40—C41—C42—C37	−0.9 (6)	C22—C23—C24—C19	−1.5 (7)
Cl9—C41—C42—C37	179.9 (3)	Cl5—C23—C24—C19	177.4 (3)
N5—C37—C42—C41	−178.7 (4)	N3—C19—C24—C23	178.6 (4)
C38—C37—C42—C41	1.8 (6)	C20—C19—C24—C23	1.0 (7)
C37—N5—C43—O9	0.1 (7)	C19—N3—C25—O5	2.3 (7)
C37—N5—C43—C44	179.5 (4)	C19—N3—C25—C26	−178.9 (4)
C52—N6—C46—C51	−168.7 (4)	C16—N2—C10—C11	9.2 (7)
C52—N6—C46—C47	12.2 (7)	C16—N2—C10—C15	−171.0 (4)
N6—C46—C47—C48	179.7 (4)	C15—C10—C11—C12	−1.8 (7)
C51—C46—C47—C48	0.7 (6)	N2—C10—C11—C12	178.0 (4)
C46—C47—C48—C49	0.6 (7)	C10—C11—C12—C13	1.2 (7)
C46—C47—C48—Cl11	−179.0 (3)	C10—C11—C12—Cl4	−179.3 (3)
C54—O12—C49—C50	−90.5 (5)	C18—O4—C13—C12	−92.0 (5)
C54—O12—C49—C48	93.6 (5)	C18—O4—C13—C14	91.8 (5)
C47—C48—C49—O12	174.5 (4)	C11—C12—C13—O4	−175.9 (4)
Cl11—C48—C49—O12	−6.0 (6)	Cl4—C12—C13—O4	4.7 (6)
C47—C48—C49—C50	−1.7 (6)	C11—C12—C13—C14	0.4 (7)
Cl11—C48—C49—C50	177.9 (3)	Cl4—C12—C13—C14	−179.0 (4)
O12—C49—C50—C51	−174.6 (4)	O4—C13—C14—C15	174.8 (4)
C48—C49—C50—C51	1.5 (6)	C12—C13—C14—C15	−1.6 (7)
O12—C49—C50—Cl12	2.6 (6)	O4—C13—C14—Cl3	−5.0 (6)
C48—C49—C50—Cl12	178.7 (3)	C12—C13—C14—Cl3	178.6 (4)
C49—C50—C51—C46	−0.3 (7)	C13—C14—C15—C10	1.1 (7)
Cl12—C50—C51—C46	−177.5 (3)	Cl3—C14—C15—C10	−179.1 (3)
N6—C46—C51—C50	−180.0 (4)	C11—C10—C15—C14	0.7 (7)
C47—C46—C51—C50	−0.8 (6)	N2—C10—C15—C14	−179.1 (4)
C46—N6—C52—O11	−2.7 (7)	C10—N2—C16—O3	−1.3 (7)
C46—N6—C52—C53	179.1 (4)	C10—N2—C16—C17	179.4 (4)

### N-(3,5-Dichloro-4-methoxyphenyl)acetamide Hydrogen-bond geometry (Å, º)

**Table:**

*D*—H···*A*	*D*—H	H···*A*	*D*···*A*	*D*—H···*A*
N1—H1*N*···O5	0.90 (2)	2.08 (2)	2.932 (4)	158 (4)
C2—H2···O1	0.95	2.29	2.885 (5)	120
C6—H6···O5	0.95	2.63	3.406 (6)	140
C8—H8*B*···Cl8	0.98	2.92	3.842 (5)	157
C9—H9*B*···O4	0.98	2.52	3.437 (6)	157
N5—H5*N*···O11^i^	0.90 (2)	2.03 (2)	2.922 (4)	174 (5)
C38—H38···O9	0.95	2.29	2.878 (6)	120
C44—H44*C*···Cl10^ii^	0.98	2.90	3.780 (5)	150
C45—H45*B*···O12	0.98	2.46	3.312 (5)	146
N6—H6*N*···O9^iii^	0.90 (2)	2.04 (2)	2.933 (4)	172 (5)
C47—H47···O11	0.95	2.32	2.899 (6)	118
C51—H51···O9^iii^	0.95	2.63	3.409 (5)	139
C53—H53*B*···Cl12^iii^	0.98	2.89	3.829 (5)	161
C53—H53*C*···O11^iv^	0.98	2.71	3.291 (5)	119
C54—H54*B*···O10^v^	0.98	2.59	3.443 (6)	145
N4—H4*N*···O3^vi^	0.90 (2)	2.03 (2)	2.924 (4)	170 (5)
C29—H29···O7	0.95	2.34	2.915 (6)	118
C33—H33···O3^vi^	0.95	2.61	3.389 (5)	140
C35—H35*B*···O1^vii^	0.98	2.57	3.311 (5)	133
C36—H36*C*···O6^vi^	0.98	2.51	3.438 (6)	157
N3—H3*N*···O1^viii^	0.89 (2)	2.08 (2)	2.910 (4)	155 (4)
C20—H20···O5	0.95	2.26	2.862 (6)	120
C26—H26*A*···O1^viii^	0.98	2.51	3.393 (5)	150
C26—H26*B*···Cl4	0.98	2.87	3.771 (5)	154
C26—H26*C*···O7	0.98	2.57	3.291 (6)	131
C27—H27*A*···Cl10	0.98	2.97	3.604 (5)	123
C27—H27*C*···O8	0.98	2.39	3.304 (6)	154
N2—H2*N*···O7^ix^	0.89 (2)	2.02 (2)	2.900 (4)	169 (4)
C11—H11···O3	0.95	2.24	2.860 (6)	122
C17—H17*A*···O7^ix^	0.98	2.56	3.391 (5)	143
C17—H17*C*···Cl6	0.98	2.83	3.772 (5)	162
C18—H18*B*···O2^v^	0.98	2.47	3.319 (6)	144

Symmetry codes: (i) −*x*+1, −*y*+2, −*z*+1; (ii) −*x*+1, −*y*+1, −*z*+1; (iii) −*x*+2, −*y*+1, −*z*+1; (iv) −*x*+2, −*y*+2, −*z*+1; (v) *x*+1, *y*, *z*; (vi) *x*−1, *y*, *z*; (vii) *x*, *y*−1, *z*; (viii) *x*+1, *y*−1, *z*; (ix) *x*, *y*+1, *z*.
